# Heme Oxygenase-1 Prevents Cardiac Dysfunction in Streptozotocin-Diabetic Mice by Reducing Inflammation, Oxidative Stress, Apoptosis and Enhancing Autophagy

**DOI:** 10.1371/journal.pone.0075927

**Published:** 2013-09-24

**Authors:** Yanli Zhao, Lina Zhang, Yu Qiao, Xiaoling Zhou, Guodong Wu, Lujing Wang, Yahui Peng, Xingli Dong, Hui Huang, Lining Si, Xueying Zhang, Lei Zhang, Jihong Li, Wei Wang, Lingyun Zhou, Xu Gao

**Affiliations:** 1 Department of Biochemistry, Harbin Medical University, Harbin, Heilongjiang, China; 2 Department of Biochemistry, Medical College of Qinghai University, Xining, Qinghai, China; 3 Key Laboratory of Cardiovascular Medicine Research, Ministry of Education, Harbin Medical University, Harbin, Heilongjiang, China; 4 State-Province Key Laboratories of Biomedicine-Pharmaceutics of China, Harbin Medical University, Harbin, Heilongjiang, China; 5 Department of Critical-Care Medicine, Affiliated Hospital of Medicine School of Qinghai University, Xining, Qinghai, China; 6 Department of Clinical Laboratory, Daqing Oilfield General Hospital, Daqing, Heilongjiang, China; Boston University School of Medicine, United States of America

## Abstract

Heme oxygenase-1 (HO-1) has been implicated in cardiac dysfunction, oxidative stress, inflammation, apoptosis and autophagy associated with heart failure, and atherosclerosis, in addition to its recognized role in metabolic syndrome and diabetes. Numerous studies have presented contradictory findings about the role of HO-1 in diabetic cardiomyopathy (DCM). In this study, we explored the role of HO-1 in myocardial dysfunction, myofibril structure, oxidative stress, inflammation, apoptosis and autophagy using a streptozotocin (STZ)-induced diabetes model in mice systemically overexpressing HO-1 (Tg-HO-1) or mutant HO-1 (Tg-mutHO-1). The diabetic mouse model was induced by multiple peritoneal injections of STZ. Two months after injection, left ventricular (LV) function was measured by echocardiography. In addition, molecular biomarkers related to oxidative stress, inflammation, apoptosis and autophagy were evaluated using classical molecular biological/biochemical techniques. Mice with DCM exhibited severe LV dysfunction, myofibril structure disarray, aberrant cardiac oxidative stress, inflammation, apoptosis, autophagy and increased levels of HO-1. In addition, we determined that systemic overexpression of HO-1 ameliorated left ventricular dysfunction, myofibril structure disarray, oxidative stress, inflammation, apoptosis and autophagy in DCM mice. Furthermore, serine/threonine-specific protein kinase (Akt) and AMP-activated protein kinase (AMPK) phosphorylation is normally inhibited in DCM, but overexpression of the HO-1 gene restored the phosphorylation of these kinases to normal levels. In contrast, the functions of HO-1 in DCM were significantly reversed by overexpression of mutant HO-1. This study underlines the unique roles of HO-1, including the inhibition of oxidative stress, inflammation and apoptosis and the enhancement of autophagy, in the pathogenesis of DCM.

## Introduction

The epidemic of obesity and a sedentary lifestyle is projected to result in over 300 million people with diabetes mellitus by 2025 [1]. One of the major causes of increased morbidity and mortality in patients with diabetes is cardiovascular complications [2]. Different pathophysiological stimuli are involved in the development of diabetic cardiomyopathy (DCM) and mediate tissue injury, leading to left ventricular (LV) systolic and diastolic dysfunction. The mechanisms of DCM are multifaceted, involving modified action potential, Ca^2+^ transient and Ca^2+^ sensitivity of contractile elements [3-5], increased oxidative stress [6-8], activation of various pro-inflammatory and apoptotic signaling pathways [9-12], decreased autophagy [13-15] and the accumulation of advanced glycation end products [16,17] among many others.

Numerous enzymes that contribute to myocardial injury have been documented to be abnormally expressed in the diabetic myocardium [8,18,19]. Heme oxygenase (HO)-1 is among these enzymes that increase in patients with diabetes [20,21]. HO, the rate-limiting enzyme in heme degradation, catalyses the oxidation of heme to generate several biologically active molecules, such as carbon monoxide (CO), biliverdin, and ferrous ion [22]. There are three isoforms in the HO family: HO-1, HO-2, and HO-3. HO-2 is constitutively expressed in most tissues. HO-3 has a similar protein structure to HO-2 but with lower enzymatic activity and is less well characterized. Whereas HO-1 is normally expressed at a low level in most tissues except for the spleen, it is highly inducible in response to a variety of stimuli (such as hydrogen peroxide, UV irradiation, endotoxins, and hypoxia) to protect cells against oxidative and inflammatory injury [23]. Numerous studies have described a contradictory role for HO-1 in the cardiovascular complications of diabetes. For example, HO-1 was shown to ameliorate glucose-induced apoptosis of human microvessel endothelial cells [24]. Up-regulation of HO-1 decreases oxidant production and endothelial cell damage and shedding and may attenuate vascular complications in diabetes [25]. Recently Cao et al. demonstrated that induction of HO-1 by treatment with cobalt-protoporphyrinIX (CoPP) improved both cardiac function and coronary flow by blunting oxidative stress [26]. However, it was also reported that HO-1 induction under hyperglycemic conditions may lead to oxidative DNA and protein damage in human umbilical vein endothelial cells (HUVECs) [27]. Another group has shown that diabetes-induced oxidative stress in the heart is in part due to increased HO-1 expression and activity, which may be mediated by an increased level of redox-active iron [28]. Thus, the role of HO-1 in the cardiovascular complications of diabetes is still uncertain.

In the present study we sought to clarify the pathophysiological functions of HO-1 in the development of DCM using wild-type HO-1 or mutant HO-1 transgenic mice. The results indicate that HO-1 activation is beneficial in preventing cardiac dysfunction and myofibril structure disarray by reducing cardiac oxidative stress, inflammation and apoptosis and enhancing cardiac autophagy.

## Materials and Methods

### Animals and Ethics Statement

All animal experiments were approved by the Institutional Animal Care and Use Committee of Harbin Medical University (No. HMUIRB-2008-06). Mice housed under identical conditions were allowed free access to a standard diet and tap water with a 12 h light: 12 h dark cycle. Mice systemically overexpressing HO-1 (Tg-HO-1) or mutant HO-1 (Tg-mutHO-1) were generated by pronuclear microinjection of fertilised eggs from C57BL/6 F1 parents with a transgenic construct expressing the cDNA for mouse HO-1 and the Gly143 to His (G143H) mutant of HO-1 under the control of the chicken β-actin promoter [29]. Transgenic mice were identified using analysis of tail DNA by PCR with a forward primer from the chicken β-actin promoter (5’-GCCTTCTTCTTTTTCCTACAGCTC-3’) and a reverse primer from the mouse HO-1 cDNA (5’-GGCATGCTGTCGGGCTGTGGAC-3’). Compared with wild-type mice, the level of the HO-1 but not the HO-2 protein in the hearts of Tg-HO-1 mice significantly increased by 5-fold [30]. In Tg-mutHO-1 mice, the G143H mutant of HO-1 binds heme but has no HO catalytic activity [31]. Male Tg-HO-1 and Tg-mutHO-1 (8 weeks of age) and their non-transgenic male littermates (Wt) were used.

### Establishment of a Mouse Model of Experimental Diabetes

Diabetes was induced in Tg-HO-1 (HO-1/DM), Tg-mutHO-1 (mutHO-1/DM) and Wt (Wt/DM) mice through consecutive peritoneal injections of streptozotocin (STZ; 50 mg/kg per day) for 5 days. After the last injection of STZ, the whole blood of all mice was obtained from the tail vein, and the glucose level in the blood was measured at random using the OneTouch Ultra 2 blood glucose monitoring system (LifeScan, Milpitas, CA). Mice were considered diabetic and used for the study only if they were hyperglycemic (>20 mmol/L) [32]. Citrate buffer-treated mice were used as non-diabetic controls (Wt/Con; blood glucose<12 mmol/L). None of the animals received exogenous insulin. After the induction of diabetes for 2 months, mice (n = 8-12 in each group) were sacrificed for the following experiments.

### Echocardiography Measurements

Two months after the induction of diabetes, echocardiographic studies were performed under sedation with pentobarbitone sodium (60 mg/kg of body weight i.p.), using an echocardiography machine (8.5-MHz linear transducer; EnVisor C, Philips Medical Systems). M-mode tracings derived from the short axis of the LV were recorded to measure LV end-diastolic diameter (LVEDD), end-systolic diameter (LVESD), left ventricular ejection fraction (LVEF), heart rate (HR) and cardiac output (CO). All measurements represent the mean of 5 consecutive cardiac cycles. The values of LVESV, LVEDV, LVEF and CO were calculated using computer algorithms.

### Blood Sample and Tissue Collection

After the assessment of LV performance, blood samples were collected from the right ventricle and the serum was separated. Serum total cholesterol (TC), triglycerides (TG) and insulin were analyzed using enzymatic methods with an automatic analyzer (JCA-BM8060, JEOL Ltd, Tokyo, Japan). Hearts were excised, washed with phosphate-buffered saline (PBS), and fixed in 10% formalin. Hearts were then transversely cut close to the apex to visualize the left and right ventricles. Four micron sections of the heart were prepared, stained with hematoxylin and eosin (H&E), visualized by light microscopy and photographed. For each sample, all available fields (>30 fields) were measured, including the septum and the right and left ventricles.

At a low temperature, a specimen removed from the left ventricular myocardium with ophthalmic scissors was cut into a 1 mm tissue mass. Images were taken after fixation, soaking, stepwise alcohol dehydration, displacement, embedding, polymerization, sectioning, and staining, and observed with an electron microscope (JEM-2000EX TEM, Tokyo, Japan). Random sections were taken and analyzed by two technicians blinded to the treatments.

### Quantitative Reverse Transcription PCR (qRT-PCR)

Total RNA was isolated with an RNA extraction kit (Axygen, CA, USA) according to the manufacturer’s protocol, and the concentration of total RNA was measured with a NanoDrop 2000c (Thermo, Fisher, MA, USA). RNA (1 µg) was converted to cDNA using reverse transcriptase (Promega, Madison, USA). RT-PCR was performed using SYBR Premix Ex Taq (Takara Bio, Tokyo, Japan).

### Western Blot Analysis

After rinsing with PBS, heart tissues or cells were lysed on ice for 30 min in lysis buffer containing a protease inhibitor cocktail (Roche, MD, USA). After centrifugation at 12000 g for 15 min, the supernatant was separated and stored at -80°C until use. Protein concentration was determined using a BCA protein assay kit (Applygen, Beijing, China). Extracted proteins were separated by sodium dodecyl sulphate polyacrylamide gel electrophoresis (SDS-PAGE), electrotransferred and immobilised on a nitrocellulose membrane. The membrane was blocked with 5% non-fat milk in PBS containing 0.1% Tween 20 (PBS-T) and incubated for 2 h. Membranes were probed with antibodies against p-Akt, p-GSK3, p-AMPK, p53, Beclin-1, LC3- II (1:1000 dilution, Cell Signaling Technology, CA, USA) or Bcl-2 (1:500 dilution, Santa Cruz Biotechnology, CA, USA). After incubation with the appropriate secondary antibody, the signal was detected using an Enzymatic Chemiluminescence (ECL) kit (Applygen, Beijing, China), and band intensities for each individual protein were quantiﬁed by densitometry, corrected for background staining, and normalized to the signal for GAPDH (1:4000 dilution, Cell Signaling Technology, CA, USA). One of three independent experiments with consistent results is shown.

### TUNEL Staining

To analysis apoptosis, terminal deoxynucleotidyl transferase-mediated dUTP nick end-labeling (TUNEL) assays were performed on sectioned mid-LV samples using an In Situ Apoptosis Detection Kit (Roche, MD, USA) according to the manufacturer’s instructions. The apoptotic index (AI) = number of TUNEL-positive myocytes / total number of myocytes stained with DAPI from a total of 40 fields per heart (n=4).

### Cell Culture

The rat embryonic heart–derived cell line H9c2 was obtained from the cell bank of the Chinese Academy of Sciences (Shanghai, China). The cells were identified by morphology and immunohistochemistry of heavy-chain cardiac myosin and alpha-cardiac actin. H9c2 cells were maintained in Dulbecco’s modiﬁed Eagle’s medium (DMEM) supplemented with 10% fetal bovine serum at 37°C in a humidified incubator containing 5% CO_2_, as described [33]. H9c2 cells stably transfected with the human HO-1 recombinant plasmid or empty vector are referred to as H9c2/HO-1 or H9c2/PC, respectively. Stably transfected clones were obtained by G418 sulphate selection. For the analysis of high-glucose treatment, cells were cultured in DMEM containing 5.5 mM glucose (control group) or 25 mM glucose (high glucose).

### Measurement of Intracellular Reactive Oxygen Species (ROS) Levels

The generation of ROS in the cells was evaluated using a fluorometry assay with the intracellular oxidation of dichlorodihydrofluorescein diacetate (DCFH-DA) (Beyotime Institute of Biotechnology, Beijing, China). After exposure to high-glucose environment for 48 h, H9c2 cells were trypsinized and centrifuged. Cell pellets were incubated with DCFDA stain (1:1000) at 37°C for 20 min in the dark. ROS levels were measured using a flow cytometer (BD Biosciences, CA, USA) and quantified by determining the mean of fluorescence for each treatment. Three independent experiments were conducted for each condition investigated, with typically 10,000 cells analyzed per experiment.

### Malondialdehyde (MDA) Assay

The MDA levels in H9c2 cells were evaluated using an MDA assay kit (Beyotime Institute of Biotechnology, Beijing, China). H9c2 cells were incubated in a 60 mm plates for 24 h for stabilization. After exposure to high glucose for 48 h, the levels of MDA were measured according to the manufacturer’s instructions. The Levels of MDA in the experimental groups were defined as a percentage compared with that of the vehicle. Three independent experiments were conducted for each condition investigated.

### Annexin V-FITC and Propidium Iodide Staining

H9c2 cells were plated in 60 mm plates. Following incubation with the appropriate drugs, 1×10^6^ cells were collected by centrifugation. Cells were resuspended in 500 µl of 1× Binding Buffer with 5 µl of Annexin V-FITC and 10 µl of propidium iodide and incubated at room temperature for 5 min in the dark. Annexin V-FITC and propidium iodide-stained cells were analyzed by flow cytometry. Three independent experiments were conducted for each condition investigated, with typically 10,000 cells analyzed per experiment.

### Statistical Analysis

The results are expressed as the means ± SD. Statistical analysis was performed using the Student 2-tailed *t* test or one-way analysis of variance (ANOVA) and Dunnett’s post hoc test. In all cases, *P* < 0.05 was considered significant. Statistical analyses were performed using SPSS Software (Version 13.0).

## Results

### Characteristics of STZ-Induced Diabetes Mellitus in Mice

The injection of STZ induced moderate to severe hyperglycemic in three mouse genotypes including Tg-HO-1, Tg-mutHO-1 and Wt mice, whereas the blood glucose in nondiabetic mice was maintained at normal levels. As shown in Table 1, compared with Wt/Con mice, food intake, water intake and urine volume were significantly increased and body weight was decreased in Wt/DM mice; furthermore, the ratio of heart to body mass was clearly increased in Wt/DM mice. Serum concentrations of TC and TG were higher and insulin was lower in Wt/DM mice than in Wt/Con mice.

**Table 1:** Basic parameters of diabetic mouse.

Basic Parameters	Wt/Con (n=12)	HO-1/Con (n=6)	MutHO-1/Con (n=6)	Wt/DM (n=12)	HO-1/DM (n=6)	MutHO-1/DM (n=5)
Blood glucose(mmol/L)	7.6±0.3	6.4±0.2	7.3±0.3	22.8±1.1^#^	21.2±1.3^#^	23.8±0.9^#^
Water intake(ml/d)	6.1±0.2	8.3±0.4	7.3±0.5	18.3±0.9^#^	19.2±0.8^#^	18.4±1.0^#^
Food intake(g/d)	6.2±0.3	7.7±0.3	6.4±0.2	16.2±0.8^#^	16.8±0.5^#^	15.7±0.8^#^
Urine volume(ml/d)	1.1±0.08	0.8±0.05	0.9±0.04	10.2±0.4^#^	9.3±0.3^#^	11.2±0.6^#^
Body mass(g)	30.4±1.5	31.3±2.1	30.6±1.8	20.1±1.3^#^	22.6±1.1^#^	19.43±1.1^#^
Heats mass(g)	0.25±0.04	0.24±0.02	0.23±0.05	0.18±0.02^#^	0.18±0.03^#^	0.19±0.03^#^
Heart to body mass ratio(mg/g)	7.7±0.6	7.3±0.3	7.6±0.4	9.1±0.4^#^	7.95±0.5*	9.8±0.6*^#^
TC(mg/dl)	55.5±10.2	53.7±12.7	61.7±11.7	102.86±14.15^#^	97.72±11.3^#^	99.36±10.9^#^
TG(mg/dl)	52.4±12.2	50.5±10.4	57.8±11.9	91.32±8.32^#^	87.46±9.49^#^	98.21±8.19^#^
Insulin(μIU/ml)	43.55±2.23	38.31±1.84	44.32±1.57	25.95±1.91^#^	27.26±2.18^#^	24.37±1.79^#^

TC, total cholesterol; TG, triglycerides. Values are presented as mean ± SD, ^#^ *p*<0.05 vs Wt/Con, *p<0.05 vs Wt/DM.

### Cardiac Function, Pathology and Ultrastructural Changes in Diabetes

**Figure 1:**
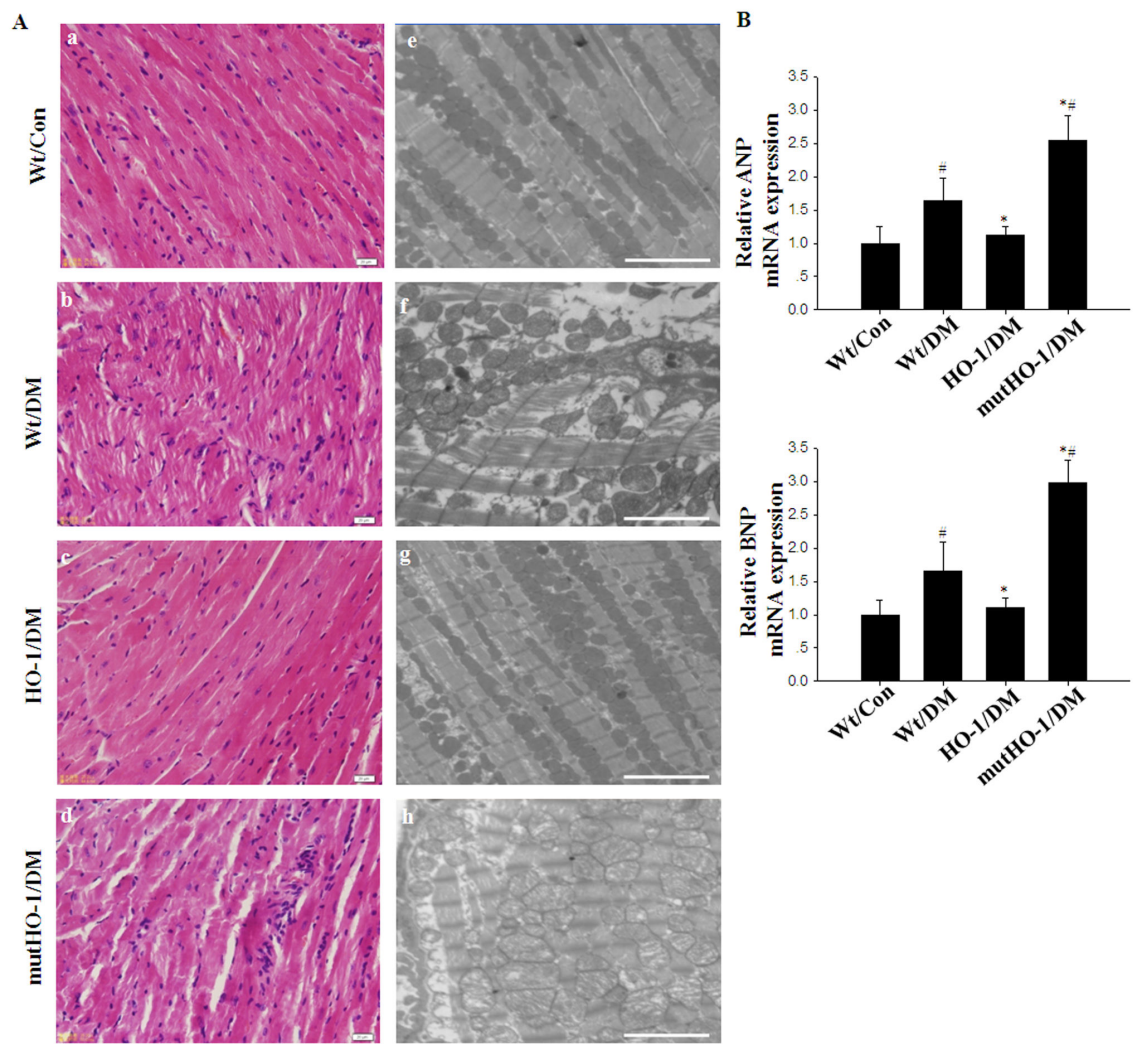
Pathology of DCM. A. Gross morphology and electron micrographs of the heart of diabetic mice. a-d: longitudinal sections of LV stained with hematoxylin and eosin (scale bar, 20µm). e-h: electron micrographs of LV (scale bars, 5 µm). B. qRT-PCR was performed to measure the levels of ANP and BNP mRNA expression in the myocardium in the various groups. GAPDH was used as an internal loading control. (n = 5 in each group) Columns and errors bars represent the mean ± SD. *#p<0.01 vs.* Wt/Con; *p<0.01 vs. Wt/DM.

The values of LVESV, LVEDV, LVEF, HR and CO were evaluated by echocardiography two months after STZ injection. Decreased LVEF and increased LVESV and LVEDV were observed in Wt/DM mice compared with Wt/Con mice as shown in Table 2. HR and CO were lower in Wt/DM mice than in Wt/Con mice. However, there was no significant difference between the two groups (Table 2). Several studies have reported that elevated expression of ANP and BNP are sensitive indicators of cardiac dysfunction [34]. In agreement with the previous studies, the expression of ANP and BNP mRNA was increased in the diabetic myocardium (Figure 1B). H&E staining and electron microscopy of the heart tissues revealed significant mitochondrial disruption and myofibril disarray in Wt/DM mice (Figure 1A). Beginning at 2-3 months after the induction of diabetes, our results are similar to the pattern of diastolic and systolic dysfunction in humans [35].

**Table 2:** Left ventricular function evaluation by echocardiograpy.

Parameters	Wt/Con (n=12)	HO-1/Con (n=6)	MutHO-1/Con (n=6)	Wt/DM (n=11)	HO-1/DM (n=6)	MutHO-1/DM (n=5)
HR(beats/min)	599±13.2	587±17.8	593±12.8	556±11.9	580±13.7	584±18.5
CO(ml/min)	1.67±0.05	1.71±0.07	1.69±0.09	1.47±0.07	1.54±0.06	1.48±0.05
LVESV(ml)	1.6±0.2	1.8±0.5	2±0.5	2.23± 0.3^#^	1.9±0.4*^#^	4.6±0.8*^#^
LVEDV(ml)	2.8±0.5	2.9±0.6	3.1±0.8	3.3±0.6 ^#^	3.29 ±0.5^#^	3.3±0.8^#^
LVEF(%)	80±6.3	82±5.7	79±5.2	68±5.9^#^	79±7.2*	60±6.8^#^

HR, heart rate; CO, cardiac output; LVESV, left ventricular end-systolic volume; LVEDV, left ventricular end-diastolic volume; LVEF, left ventricular ejection fraction.

Values are presented as mean ± SD, ^#^ *p*<0.05 vs Wt/Con, *p<0.05 vs Wt/DM.

### Up-Regulation of HO-1 Expression in the Hearts of Mice with Diabetes Mellitus

**Figure 2:**
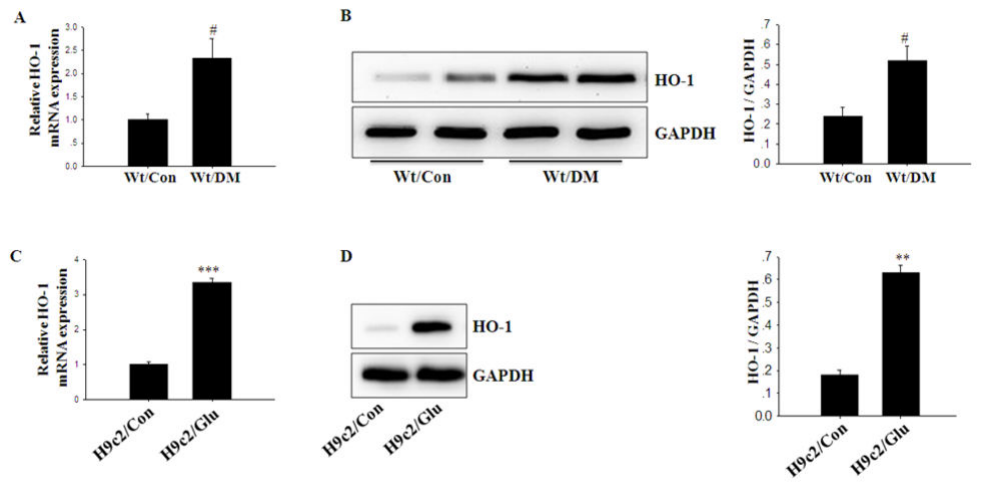
Enhanced HO-1 expression in diabetic hearts. A, B. After two months of established diabetes, mice were killed, and left ventricles of the heart were excised. HO-1 levels in samples were analyzed by qRT-PCR and western blot. GAPDH was used as an internal loading control. (n = 5 in each group). C, D. After a 48 h incubation with high-glucose, HO-1 expression in H9c2 cells was analyzed by qRT-PCR and western blot. GAPDH was used as an internal loading control. Columns and errors bars represent mean ± SD. *** *p<0.001 vs.* H9c2/Con; *#p<0.01 vs.* Wt/Con.

Cardiac HO-1 expression was notably increased in Wt/DM mice compared with Wt/Con mice as shown in Figure 2(A, B). In H9c2 cells, high glucose also induced a significantly enhanced expression of HO-1 (Figure 2C, D).

Improved diabetes-induced LV dysfunction and myofibril structure disarray in HO-1/DM mice.

To evaluate whether HO-1 mediated cardioprotective effects, transgenic mice systemic overexpressing HO-1 were utilized in the following study [14,15]. Compared with Wt/DM mice, the heart to body mass ratio was decreased in HO-1/DM mice. However, there were no differences in the food intake, water intake, urine volume, TC, TG or insulin between Wt/DM and HO-1/DM mice (Table 1). Moreover, in HO-1/DM mice, LVESV was decreased, and LVEF was increased (Table 2). H&E staining and electron microscopy revealed a greater attenuation of the mitochondria disruption and myofibril disarray than in Wt/DM mice (Figure 1A). Concurrently, HO-1/DM mice showed significantly down-regulated expression of ANP and BNP compared with Wt/DM mice (Figure 1B). Over all, these results indicate that HO-1 improves diabetes-induced LV dysfunction and myofibril structure disarray in diabetic mice.

### Attenuated Diabetes-Induced Myocardial Oxidative Stress and Inflammation in HO-1/DM Mice

**Figure 3:**
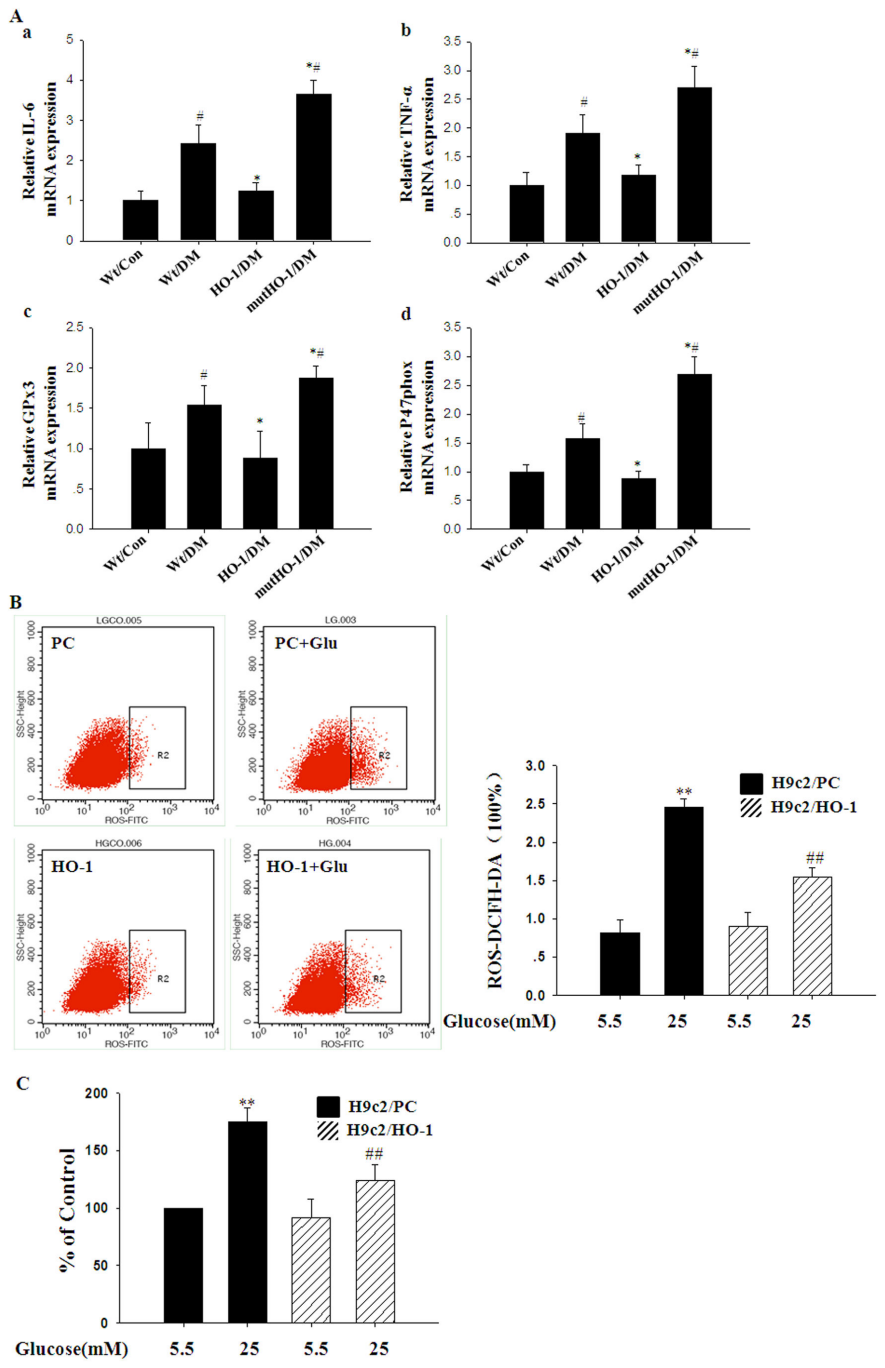
Anti-inflammatory and anti-oxidant effects of HO-1 on cardiomyocytes in diabetic mice. A. LV expression of IL-6 (a), TNF-a (b), GPx3 (c), P47phox (d) mRNA in the respective groups. GAPDH was used as an internal loading control (n = 5 in each group). B, C. Quantification of ROS and MDA in HO-1 or empty vector (PC) transfected H9c2 cells under control and high-glucose culture conditions for 48 h. Columns and errors bars represent mean ± SD. *#p<0.01 vs.* Wt/Con; *p<0.01 vs. Wt/DM; ** *p<0.01 vs.* PC/Con; *##p<0.01 vs.* PC/Glu.

We further investigated the underlying mechanism by which HO-1 protected against diabetic cardiomyopathy. Increased oxidative stress may result in the formation of cell-damaging gene products, which then lead to various diabetic vascular complications [36,37]. Our results indicated that the expression of NADPH oxidase subunit p47phox (p47phox) and glutathione peroxidase-3 (GPx3) mRNA was remarkably elevated in Wt/DM mice compared with Wt/Con mice. Overexpression of HO-1 decreased the expression of p47phox and GPx3 mRNA compared with Wt/DM mice (Figure 3 A c, d). These results were in accordance with the presence of oxidative stress, characterized by the accumulation of ROS and MDA in H9c2 cells under high glucose. ROS and MDA levels significantly increased in H9c2 cells incubated with high glucose. However, overexpression of HO-1 significantly decreased ROS and MDA levels in H9c2 cells cultured in high-glucose conditions (Figure 3B, 3C).

Cardiac inflammation characterized by increased expression of pro-inflammatory cytokines plays an important role in the pathophysiology of DCM [12,38,39]. In the present study, LV expression of interleukin-6 (IL-6) and tumor necrosis factor-α (TNF-α) mRNA was notably increased in Wt/DM mice compared with Wt/Con mice. Overexpression of HO-1 decreased the expression of IL-6 and TNF-α mRNA compared with Wt/DM mice (Figure 3 A a, b).

### Attenuation of Cardiac Apoptosis and Restoration of Cardiac Autophagy in HO-1/DM Mice

**Figure 4:**
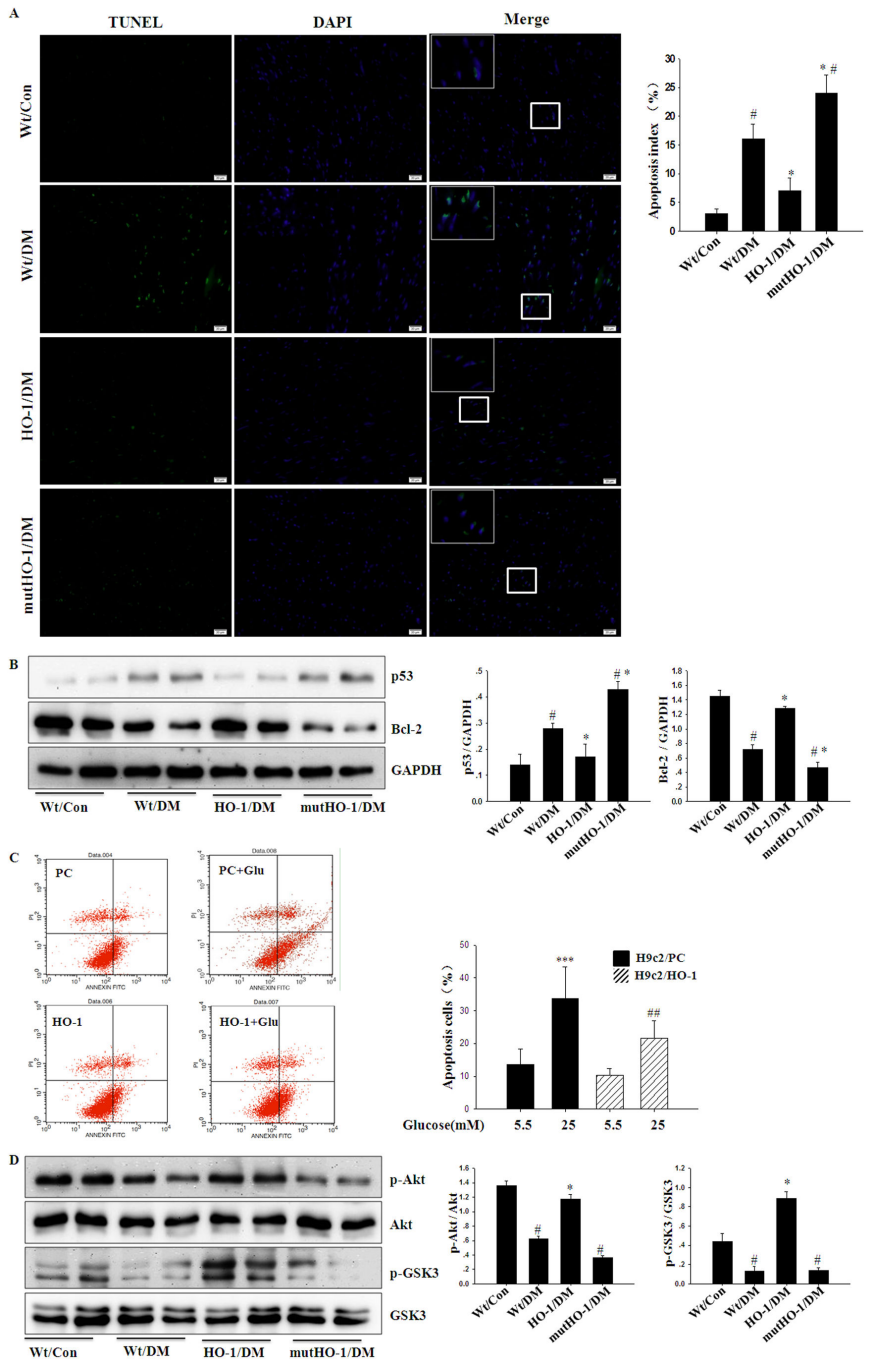
Anti-apoptotic effects of HO-1 on cardiomyocytes in diabetic mice. A. Apoptosis assessed by TUNEL assay in the respective groups (left) with quantification on the right. First column: Nuclei of apoptotic cells TUNEL stained in green. Second column: The DAPI-stained nuclei appear in blue. Third column: merged picture of first and second column (scale bar, 20µm). B. Representative immunoblot for p53 and Bcl-2 in the myocardial tissues from the respective groups (left) and densitometric quantification (right). C. Flow cytometric analysis using Annexin V-FITC and propidium iodide stains to evaluate the effects of transfection with HO-1 or empty vector (PC) under control and high-glucose culture conditions (left) with quantification on the right. D. Representative immunoblot for p-Akt, Akt, p-GSK3 and GSK3 in the myocardial tissues from the respective groups (left) and densitometric quantification (right). (n = 5 in each group) Columns and errors bars represent mean ± SD. *#p<0.01 vs.* Wt/Con; *p<0.01 vs. Wt/DM; *** *p<0.001 vs.* PC/Con; *##p<0.01 vs.* PC/Glu.

Apoptosis is reported to play a critical role in DCM [40,41]. In heart sections generated from HO-1/DM mice, we found that the number of TUNEL-positive cells was signiﬁcantly decreased compared with those detected in Wt/DM mice (Figure 4A). In H9c2 cells, overexpression of HO-1 strongly inhibited 25 mM glucose-induced apoptosis as assessed by flow cytometry of AnnexinV-FITC and propidium iodide-stained cells (Figure 4C). Immunoblotting revealed that the expression of p53 was decreased and Bcl-2 was markedly increased in HO-1/DM mice compared with Wt/DM mice (Figure 4B). Furthermore, we tested the hypothesis that the anti-apoptotic effect of HO-1 was mediated by activation of the Akt pathway. Compared with Wt/Con, the phosphorylation of Akt and GSK-3 was decreased in Wt/DM mice. These changes were attenuated in HO-1/DM mice, suggesting that the Akt pathway may be involved in the crucial role of HO-1 in DCM (Figure 4D). These results indicate that the cardio-protective effects of HO-1 may be mediated in part by the attenuation of cardiac apoptosis via Akt activation in diabetic mice.

**Figure 5:**
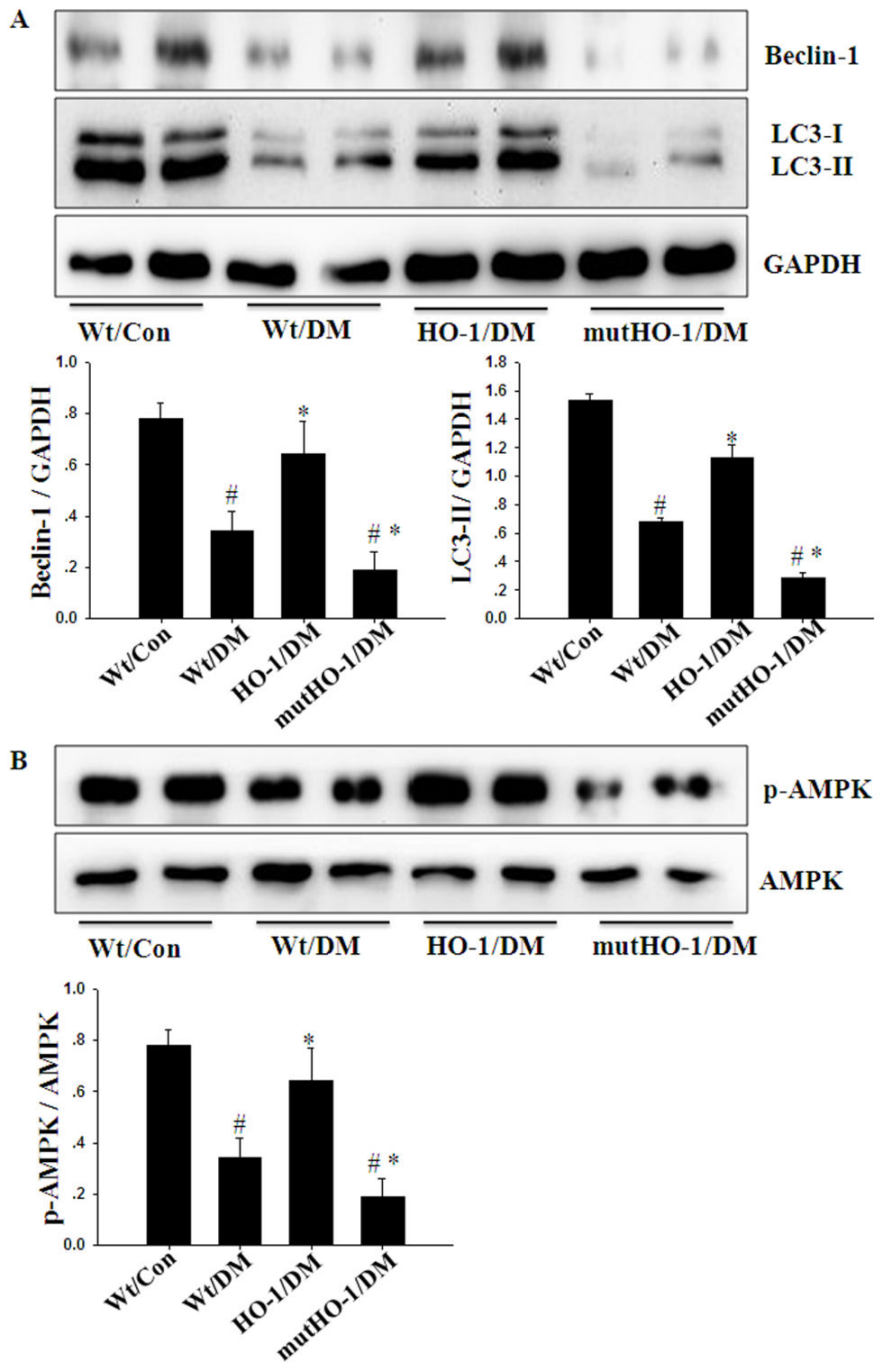
The protective effect of HO-1 on myocardial autophagy in diabetic mice. A, B. Representative immunoblot for Beclin-1, LC3-II, p-AMPK and AMPK in the myocardial tissues from the respective groups (above) and densitometric quantification (below). (n = 5 in each group). Columns and errors bars represent mean ± SD. *#p<0.01 vs.* Wt/Con; *p<0.01 vs. Wt/DM.

A basal level of autophagy plays an important role in protecting cardiomyocytes from hyperglycemic damage, and the suppression of autophagy in diabetes contributes to the development of cardiomyopathy [42]. To assess the role of autophagy in DCM, we measured the expression of cardiac LC3-II and Beclin-1 after two months of diabetes. Remarkably, the expression of cardiac LC3-II and Beclin-1 was decreased in Wt/DM mice compared with Wt/Con mice. Overexpression of HO-1 abrogated the reduced LC3-II and Beclin-1 expression in diabetic hearts (Figure 5A). Furthermore, emerging evidence demonstrated that AMPK may regulate autophagy in diabetic cardiomyopathy [43]. Thus, we also examined phosphorylation of AMPK in diabetic mice. As shown in Figure 5B, the phosphorylation of AMPK was decreased in Wt/DM compared with Wt/Con mice. Overexpression of HO-1 increased AMPK induction, suggesting that the cardio-protective effect of HO-1 at least partially underlies the restoration of cardiac autophagy via AMPK activation in diabetic mice.

### Impairment of Cardiac Function, Promotion of Cardiac Inflammation, Oxidative Stress and Apoptosis, and Suppression of Autophagy in mutHO-1/DM Mice

To further investigate our hypothesis that HO-1 plays a cardioprotective role in DCM, we generated Tg-mutHO-1 mice in which Gly143 was mutated to His [13]. In mutHO-1/DM mice, the heart to body mass ratio (Table 1) and LVESV (Table 2) were increased compared with Wt/DM mice. Cardiac pathology and ultrastructural changes were exacerbated (Figure 1A), and the expression of ANP and BNP mRNA was significantly up-regulated in mutHO-1/DM mice compared with the Wt/DM mice (Figure 1B). Overexpression of mutant HO-1 markedly increased the expression of IL-6, TNF-α, p47phox, and GPx3 mRNA in diabetic mice (Figure 3). In heart sections from mutHO-1/DM mice, the number of TUNEL-positive cells was signiﬁcantly increased compared with Wt/DM mice (Figure 4A). Immunoblotting showed that the expression of p53 was increased and Bcl-2 was decreased in mutHO-1/DM mice. Concurrently, phosphorylation of Akt but not GSK-3 was lower in mutHO-1/DM mice than in Wt/DM mice (Figure 4D). Molecular markers of autophagy in the hearts of mutHO-1/DM mice were markedly decreased as indicated by LC3-II and Beclin-1 protein levels (Figure 5A). The phosphorylation of AMPK was also decreased in mutHO-1/DM mice compared with Wt/DM mice (Figure 5B).

## Discussion

**Figure 6:**
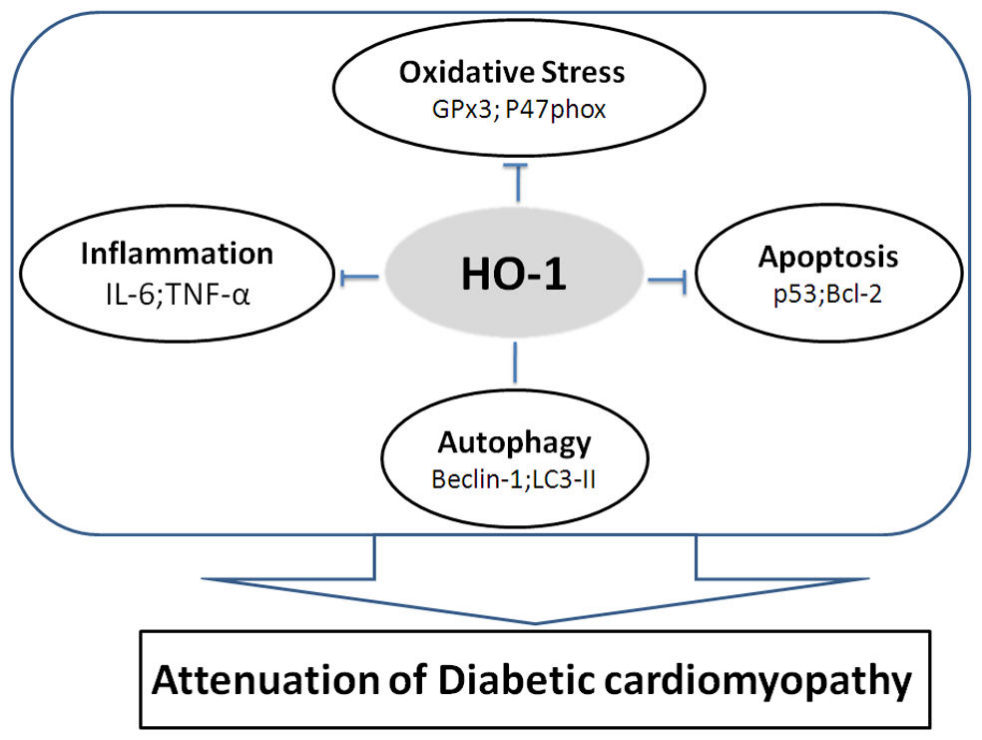
Scheme for the possible mechanism for the attenuation of diabetic cardiomyopathy by heme oxygenase-1 (HO-1) overexpression.

To our knowledge, the present study is the firsr to describe that overexpression of HO-1 protects against cardiac dysfunction and attenuates mitochondrial disruption and myofibril disarray in DCM. The effect of HO-1 in DCM is associated with the attenuation of myocardial oxidative stress, inflammation and apoptosis and enhancement of autophagy (Figure 6). In contrast, overexpression of mutant HO-1 is not cardioprotective.

DCM is defined as the ventricular dysfunction that occurs in diabetic patients independently of coronary artery disease or hypertension [2,44]. Our results demonstrated that STZ injection successfully induced diabetes and DCM as indicated by an increase in cardiac dysfunction and myocardial structure changes. These cardiac abnormalities were improved by the overexpression of HO-1 and were exacerbated by the overexpression of mutant HO-1. These findings suggest that HO-1 represent a realistic strategy for limiting the progression of cardiac dysfunction associated with diabetes.

Accumulating evidence suggests that increased oxidative stress coupled with the activation of various downstream pro-inflammatory and apoptotic pathways plays a ivotal role in the development of complex biochemical, mechanical and structural alterations associated with DCM [2,45-47]. HO-1 is a stress-response protein that activated under conditions of increased oxidative stress [48,49]. Cao et al. reported that up-regulating HO-1 improves cardiac and vascular dysfunction by decreasing oxidative stress in hypertensive rats fed a high-fat diet [50]. Furthermore, induction of HO-1 results in decreased cardiac expression of superoxide and NOX-2, which may be due to a decrease in the levels of NADPH oxidase [51]. Interestingly, utilizing tin protoporphyrin IX, a potent inhibitor of the HO system, Farhangkhoee and colleagues demonstrated that diabetes-induced oxidative stress in the heart is due to up-regulation of HO expression and activity [27]. Most studies of HO-1 utilise chemicals that induce or inhibit HO-1. Moreover, numerous publications have shown marked adverse effects of HO-1 by HO-1 inducers or inhibitors in various in vivo and in vitro experimental models. Furthermore, treatment with an HO-1 inducer or a recombinant adenovirus carrying the HO-1 gene causes more acute stress than transgenic mice overexpressing HO-1. Thus, the results of acute stress might be different from those of long-term transgenic overexpression. We eliminated the interference from an inducer or inhibitor of HO-1 by using Tg-HO-1 and Tg-mutHO-1 mice. Recent studies have demonstrated that p47phox and GPx3, two antioxidant enzymes, are increased in DCM in response to enhanced oxidative stress [52,53]. Consistent with the above-mentioned studies, our data showed that the expression of p47phox and GPx3 mRNA was decreased in HO-1/DM mice and increased in mutHO-1/DM mice compared with Wt/DM mice. In H9c2 cells, overexpression of HO-1 significantly decreased myocardial ROS and MDA levels under high-glucose conditions. Collectively these data show that HO-1 protects against DCM at least partially by reducing oxidative stress.

Additionally, an increasing amount of experimental and clinical evidence suggests that pro-inflammatory cytokines are involved in the pathogenesis of heart dysfunction [54,55]. TNF-α is an important factor in the development and progression of heart failure [56,57]. IL-6 is involved in the development of atherosclerosis, myocardial remodeling, and experimental and clinical cardiac dysfunction [58,59]. The inflammatory cytokines IL-6 and TNF-α, are known to promote LV dysfunction and play a pathogenic role in heart failure and DCM [51,53,60]. Moreover, HO-1 plays a critical protective role during the development of inflammation in the cardiovascular system [61,62]. Mougiakakos et al. observed that pharmacological inhibition of HO-1 activity led to a significant reduction in the ability of these cells to induce regulatory T cells [63]. Similarly it has been reported that hepatic overexpression of HO-1 leads to the induction of regulatory T cells that are responsible for increased survival of liver allografts [64]. Orozco et al. also reported that decreased or absent HO-1 expression in peritoneal macrophages results in enhanced ROS formation and increased inflammatory cytokines such as MCP-1, IL-6, and the murine interleukin 8 homolog [65]. Our results from the present study also agree with previous studies demonstrating that the expression of IL-6 and TNF-α mRNA was decreased in HO-1/DM mice and increased in mutHO-1/DM mice compared with Wt/DM mice. Together these data suggest that the decreased cardiac inflammation induced by HO-1 may lead to protection from DCM.

Oxidative stress and inflammation stimulated apoptosis are reported to play a critical role in DCM [39,40]. HO-1 induction in the failing heart is an important cardioprotective adaptation that opposes pathological LV remodeling, and this effect is mediated by product-dependent inhibition of apoptosis [66]. Indeed, our results showed that the number of TUNEL-positive cells was decreased in HO-1/DM mice and increased in mutHO-1/DM mice compared with Wt/DM mice. Furthermore, the expression of p53 and Bcl-2 confirmed the results from TUNEL staining. Moreover, in H9c2 cells, overexpression of HO-1 significantly decreased the number of apoptotic cells that were induced by high glucose. The Akt/GSK-3 pathways are commonly involved in stress-induced apoptosis in vitro and in vivo models [67-69]. In fact, the Akt pathway is activated during hydrogen peroxide-induced apoptosis in H9c2 cells [70] and mesenchymal stem cells [71] as well as in in vivo models of DCM [51]. Accordingly, we demonstrated that the reduced apoptosis in HO-1/DM mice was associated with significantly increased phosphorylation of Akt and GSK-3. These changes were reversed in mutHO-1/DM mice. Therefore, our findings suggest that HO-1 protects against DCM in part by reducing apoptosis, which is regulated by Akt activation.

In the heart, constitutive autophagy is a homeostatic mechanism for maintaining cardiac structure and function [72]. Xie et al. showed that basal levels of autophagy are important for protecting cardiomyocytes from hyperglycemic damage and that the suppression of autophagy in diabetes contributes to the development of cardiomyopathy [41]. In the same investigation, it was reported that decreased AMPK activity and the subsequent reduction in cardiac autophagy are important events in the development of diabetic cardiomyopathy [13]. A recent study also suggested that the dissociation of Bcl-2 from Beclin1 may be an important mechanism for preventing diabetic cardiomyopathy via AMPK activation that restores autophagy and protects against cardiac apoptosis [14]. Furthermore, Carchman’s study reported that sepsis or LPS-induced autophagy protects against hepatocellular death, in part via an HO-1, p38-MAPK-dependent signaling pathway [73]. Indeed, our results suggest that overexpression of HO-1 enhances the phosphorylation of AMPK and increases LC3-II and Beclin-1 expression in diabetic cardiomyopathy. However, overexpression of mutant HO-1 reverses the effects of HO-1. Collectively, our findings suggest that HO-1 prevents DCM in part through AMPK activation and by up-regulating autophagy.

## Conclusions

We conclude that HO-1 plays an important role in the pathogenesis of DCM by inhibiting oxidative stress, inflammation and apoptosis and enhancing autophagy.
